# Relationships among Affect, Hardiness and Self-Efficacy in First Aid Provision by Airline Cabin Crew

**DOI:** 10.3390/ijerph18042108

**Published:** 2021-02-22

**Authors:** Yi-Chen Yu, Jyh-Chong Liang

**Affiliations:** 1 China Airlines, Taoyuan 337, Taiwan; schnauzer.yu@gmail.com; 2 Program of Learning Sciences and Institute for Research Excellence in Learning Sciences, National Taiwan Normal University, Taipei 106, Taiwan

**Keywords:** cabin crew, self-efficacy of first aid, affect, hardiness, mediation effect

## Abstract

Cabin crews being first responders, passengers’ health assurance is also one of their main responsibilities. This study explored the association among first aid affect, work-related hardiness and self-efficacy of first aid, as well as the mediation role of work-related hardiness in airline cabin crews. Three self-reporting instruments were applied in this study: one was the first aid affect questionnaire, the second was a work-related hardiness questionnaire, the third was self-efficacy of the first aid questionnaire. Data were collected from 525 cabin crew members across five airlines in Taiwan (480 females and 45 males). The results showed that both exploratory and confirmatory factor analyses indicated that three instruments had satisfactory validity and reliability. Positive significant relationships were found among cabin crews’ first aid affect, work-related hardiness and self-efficacy of first aid. Cabin crews’ commitment dimension of work-related hardiness turned out to be positively related to self-efficacy of first aid. In addition, the results of the study also revealed that cabin crews’ work commitment plays a mediating role between their first aid affect and self-efficacy of first aid. To enhance the self-efficacy of first aid, it is necessary for the airlines to strengthen cabin crews’ work commitment. Furthermore, fostering cabin crews’ first aid affect is also one an important training goal.

## 1. Introduction

### 1.1. Background

According to the International Civil Aviation Organization’s (2018) annual report [1], the global air passenger traffic has reached 4.3 billion passengers, an increase of 7.1% over the previous year. Although air travel is generally safe, the possibility of in-flight medical emergencies may increase with the growing air passenger traffic and the large passenger capacity of big commercial airliners [2,3]. Long-haul flights that travel over 16 h may cause a more significant adverse effect on the elderly or people with a pre-existing medical condition [4,5]. One study suggested that medical emergencies occur in every 1 out of 604 flights [6].

Cabin crews play an important role in dealing with aviation crisis situations. They are the first responders to ensure the safety and comfort of airline passengers. When it comes to in-flight medical emergencies, they must react immediately and conduct an initial assessment of the patient and provide pre-hospital care. Based on British Airways statistics, Dowdall [7] calculated that the occurrence rate of in-flight medical incidents is about one per 11,000 passengers, and 70% of them were handled by cabin crew. Due to the narrow cabin space and limited medical equipment on the aircraft, managing medical emergencies and resuscitation could be highly stressful to cabin crews, especially when dealing with scared patients. It requires the cabin crew not only to have first aid knowledge and skills, but also to remain calm under pressure, hold positive attitudes toward their work, and feel confident that they could perform first aid successfully. These qualities, such as cabin crew’s affective state, work-related commitment, and their self-efficacy toward first aid are the main foci of the current study.

Self-efficacy is defined as an individual’s belief concerning his/her ability to achieve goals [8]. It is considered as an important predictor of people’s academic or work performance [9]. Previous research has found that the lack of confidence made cabin crew less willing to perform first aid [10]. If cabin crew can assist the injured or sick passengers in time, they can prevent the illness from worsening or even leading to death, and reduce the cost of changing flight routes or delays. To date, there is a scarcity of studies exploring the influencing factors of cabin crews’ self-efficacy of first aid. Therefore, there is a need to investigate the predicting factors of self-efficacy in order to identify the sources of cabin crew’s first aid self-efficacy.

### 1.2. First Aid in Flight

In a recent study, Paxinos et al. [11] showed that the most common in-flight medical problems were loss of consciousness (38.4%), injuries (8.6%), gastrointestinal problems (8.3%), respiratory symptoms (7.3%), anxiety (5.7%), and burns (5.9%). Also, Valani et al. [12] reported the medical conditions most commonly leading to flight diversions were cardiac, neurological, gastrointestinal, and syncope incidents.

In order to respond to potential medical emergencies, most airlines provide a first-aid kit, a medical kit, universal precaution kits and an automatic defibrillator on board. Supplemental oxygen is also available. The cabin crew have a responsibility to manage medical emergencies in flight. Moreover, each cabin crew must receive first aid recurrent training annually, including life-threatening medical emergencies, cardiopulmonary resuscitation, management of injuries, management of illnesses, handling first-aid and medical equipment and supplies [13].

#### 1.2.1. First Aid Affect

##### Attitude

People’s attitude and willingness to perform first aid constitute their first aid affect. Believing that performing first aid is a meaningful act to self and others could lead to more involvement in first aid training or practices [14]. Breckler [15] theorized that attitude was made up of three components: affective, behavioral, and cognitive, which were only moderately correlated with each other. Moreover, Kraus [16] concluded from a meta-analysis that attitudes could significantly predict further behavior. People’s first aid affect is found to be associated with self-efficacy in first aid. For instance, research found that elementary and junior high school students who held a more positive attitude toward first aid had higher self-efficacy of first aid [17,18].

##### Willingness

Existing literature found that individuals’ willingness to perform cardiopulmonary resuscitation (CPR) or attend CPR training mainly depend on their affect toward first aid [19,20,21]. People have many reasons to feel reluctant to perform first aid, the lack of CPR skills being the first one. They are concerned that something might go wrong [22,23]. Lin et al. [10] found that only 19% of cabin crews in Taiwan were willing to perform first aid. “Do not know how to do it” and “lack of confidence in their ability” were major reasons for their reluctance to act. Besides prior first aid knowledge, the fear of potential disease transmission, physical appearance of the victim, and perceptions of their ability to perform first aid were also found to affect their willingness to administer first aid [24,25].

People’s affect toward first aid was found to be correlated with their confidence in dealing with medical emergencies. A study by Pei et al. revealed that nursing students’ willingness, knowledge, and attitudes toward first aid behavior were positively correlated with their self-efficacy of first aid [26]. Choi et al. [27] found that students with higher self-efficacy were more willing to perform CPR. Studies have also shown that individuals who had a better understanding of CPR procedures, the use of automated external defibrillator (AED), or law liability held more positive attitude and willingness to perform first aid [22,25,28,29]. Another research reported that students with prior training in basic life support, having confidence in their basic life support skills were more willing to perform first aid [30].

### 1.3. Self-Efficacy of First Aid

Self-efficacy refers to an individual’s beliefs in his/her capability to perform the tasks, and it is domain-specific [8]. In order to measure health professionals’ and students’ self-efficacy of dealing with medical emergencies, some scales of resuscitation self-efficacy have been developed [31,32,33]. Maibach et al. [34] indicated that self-efficacy could be one of the most important factors of resuscitation proficiency. For example, Roh et al. [35] determined the impact of nursing students’ self-efficacy in chest compression. Maibach et al. [34] proposed that even health professionals may fail to apply resuscitation successfully unless they have a strong belief in their capability. As Bandura [36] noted, perceived self-efficacy influences all aspects of behavior. Cabin crew’s self-efficacy of first aid is crucial for managing in-flight medical emergencies.

#### Measurement of Self-Efficacy of First Aid

Roh et al. [32] developed the Resuscitation Self-Efficacy Scale (RSES) to evaluate resuscitation self-efficacy, including four dimensions, namely “Recognition”, “Debriefing and recording”, “Responding and rescuing”, and “Reporting”. Results showed that experienced nurses reported higher self-efficacy than new graduate nurses. In order to accurately measure nursing students’ confidence levels of their capabilities when responding to a cardiac arrest, Hernández-Padilla et al. [33] developed The Basic Resuscitation Self-Efficacy Scale (BRS-SES), which revealed three dimensions: Recognition & Alertness, Cardiopulmonary Resuscitation, and Safe use of an AED. Results indicated that the BRS-SES has sound psychometric properties and good validity. In addition, the tool could contribute to the implementation and standardization of the training of basic resuscitation skills. Previous research has indicated that the lack of confidence made cabin crew less willing to perform first aid [10]. In a similar study by Mahony et al. [37], cabin crew generally held low levels of self-confidence in their CPR and AED skills, even though their confidence in first aid knowledge was high, indicating that cabin crew may not have sufficient skills to manage a cardiac arrest adequately. Therefore, it is important to explore what factors would be associated with self-efficacy of first aid in cabin crew. Based on these theoretical backgrounds, we posited that:

**Hypothesis** **1** **(H1).** First Aid Affect (Attitude and Willingness) is positively associated with Self-Efficacy of First Aid (Recognition, Debriefing and recording, Responding and rescuing, Reporting, and Safe use of AED.)

### 1.4. Hardiness

Kobasa [38] defined hardiness as a personality characteristic with continued good health and performance under stress, comprising three components: commitment, control, and challenge. The study revealed that executives high in hardiness coped effectively with stress and had less illness.

#### 1.4.1. Measurement of Hardiness in Different Contexts

To gain a better understanding of why some students were willing to pursue challenging courses, while others avoided taking difficult classes, Benishek et al. [39] created Academic Hardiness Scale (AHS) to assess hardiness in students, including three dimensions: Commitment, Control and Challenge. Hence, it indicates that hardy students have three common characteristics: they are committed to their academic work and are involved with all their courses; they believe they can control over their academic performance and outcomes, and they are willing to engage in challenging academic work and view academic challenges as learning opportunities.

However, Benishek et al. claimed that a number of limitations were associated with the AHS. So they added additional aspects, and adjusted the Control subscale into an affective-oriented and effort-oriented dimension. Therefore, the Revised Academic Hardiness Scale (RAHS) was designed to assess four dimensions: Control of affect, Control of effort, Commitment and Challenge [40]. 

#### 1.4.2. The Relationships between Hardiness and Other Variables

Fisher [41] found that computer attitudes were positively correlated with the personality hardiness. Moreover, Moradi et al. [42] also revealed that students with greater psychological hardiness and self-efficacy may have more positive attitudes toward the computer. Those studies implied that improving individual’s attitude may strengthen their hardiness.

In addition, some studies have investigated the relationship between hardiness and self-efficacy. Cheng et al. [43] claimed that academic hardiness was a strong predictor of graduate students’ academic self-efficacy. Wang et al. [44] found that both teacher’s and students’ science hardiness were associated with science learning self-efficacy. Similar results were found in the research of Vasudeva et al. [45], who revealed positive relationships between women’s hardiness and self-efficacy. Nasiri [46] performed a study on hardiness and self-efficacy with job satisfaction and showed that high school teachers with higher levels of hardiness had higher levels of self-efficacy. According to the literature, people with higher self-efficacy are more likely to set challenging goals and more committed to their duties. In this research, we seek to evaluate the association of work-related hardiness and self-efficacy of first aid. Based on the literature review, we hypothesized that:

**Hypothesis** **2** **(H2).** First Aid Affect (Attitude and Willingness) is positively associated with Work-Related Hardiness (Commitment, Control, and Challenge).

**Hypothesis** **3** **(H3).** Work-Related Hardiness (Commitment, Control, and Challenge) is positively associated with Self-Efficacy of First Aid (Recognition, Debriefing and recording, Responding and rescuing, Reporting, and Safe use of AED).

### 1.5. First Aid Affect, Work-Related Hardiness and Self-Efficacy of First Aid

Based upon the above literature review, first aid affect and hardiness were related to self-efficacy. As far as we are aware of, no previous research has investigated the association among first aid affect, hardiness, and self-efficacy of first aid.

Both the association between first aid affect and self-efficacy of first aid and the association between hardiness and self-efficacy have been confirmed separately by previous studies [27,30,43,44]. Work-related hardiness is likely to be a mediator to influence the strength or direction of the relationship between first aid affect and self-efficacy of first aid [47]. To our knowledge, the role of hardiness as a mediator between affect and self-efficacy has not been examined. In order to establish the type of mediation, we followed the procedure presented by Zhao et al. [48]. Hence, we posited that:

**Hypothesis** **4** **(H4).** The relationship between First Aid Affect and Self-Efficacy of First Aid is mediated by Work-Related Hardiness.

**Figure 1:**
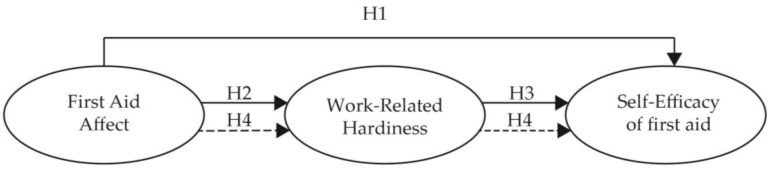
The theoretical model.

Thus, the present study aimed at exploring the structural relationships among cabin crews’ first aid affect, work-related hardiness and self-efficacy of first aid and assessing the mediating effect of work-related hardiness. Based on the previous literature review, we established a hypothetical model, as shown in Figure 1.

## 2. Methodology

### 2.1. Participants

From April to June 2017, a total of 560 surveys were distributed among employees of five airlines in Taiwan. At the beginning of the survey, we informed the participants of their right to withdraw from the study at any time, and provided clear information and instructions that might help improve future training sessions. Three questionnaires were filled out at the same time. Questionnaires with missing data were excluded, and the final sample included 525 cabin crews, with 480 females and 45 males. Since the majority of flight attendants were female, the participants represented the normal gender ratio of cabin crew. Their age ranged from 22 to 63, with an average age of 34.49. Their average tenure as cabin crew with the company was 9.9 years, and 19.9% of the participants were cabin managers/pursers, 79.8% were cabin crew members.

### 2.2. Instruments

Three separate questionnaires were used to evaluate cabin crews’ first aid affect, work-related hardiness, and first aid self-efficacy. The questionnaires were translated into Chinese in this study. All the questionnaire items were reviewed by one educational expert who had experiences in developing surveys and one aviation first aid teacher who had experiences in crew training. Brief descriptions are provided as follows.

#### 2.2.1. The Questionnaire of First Aid Affect (FAA)

The first questionnaire with two dimensions (Attitude and Willingness toward first aid) was used to assess cabin crews’ attitudes and willingness toward first aid. The attitude toward first aid dimension was adapted and modified from Lee et al.’s [20] questionnaire for measuring high school student’s attitude toward first aid in Taiwan. The original questionnaire dimension consisted of 12 items, and the internal consistency reliability coefficient alpha was reported as 0.88. We made some adjustments to the original questionnaire by deleting unrelated items. Eventually, six questionnaire items surveying cabin crews’ attitudes toward first aid were proposed, including having knowledge and skills in first aid, saving a life, preventing the injury from getting severe. The two experts decided that these items were sufficient for representing crew’s perceptions of the attitudes toward first aid. The items were measured on a 5-point Likert scale ranging from 1 “strongly disagree” to 5 “strongly agree”. An example item of this dimension is “Actively participate in first aid courses and activities.” Cabin crew who scored higher on this questionnaire were deemed to have more positive attitudes toward first aid.

The willingness toward first aid dimension was developed based on the subjects within the scope of first aid training set by the IOSA standards manual (2020) [13] and with reference to the most common in-flight medical emergencies [6,7]. This dimension contains 11 questions surveying cabin crews’ perceptions of their willingness to deal with a list of emergency situations, including using a defibrillator (AED), handling precipitate labor, asthmatic attack, hyperventilation, burn, choking, cardiopulmonary resuscitation, seizure, heart attack, stroke, and syncope. The items were reviewed by the two experts and considered sufficient for measuring the crew’s perceptions of their willingness toward first aid emergencies. For each statement, cabin crew have to mark their response on a 5-point Likert scale. Higher scores represented more positive willingness toward first aid.

#### 2.2.2. The Questionnaire of Self-Efficacy of First Aid (SEFA)

We combined two questionnaires into SEFA to measure cabin crews’ self-efficacy of first aid. The first questionnaire was the Basic Resuscitation Self-Efficacy Scale developed by Hernández-Padilla et al. [33] to measure cabin crews’ confidence levels in their capabilities in responding to a cardiac arrest. It included 18 items within three dimensions, namely Recognition & Alertness, Cardiopulmonary Resuscitation and Safe use of an AED, and used a 5-point Likert scale ranging from 1 “very unconfident” to 5 “very confident.” The reliability coefficient alpha was 0.96 for the total and 0.85, 0.92, and 0.96, for the three subscales respectively. The second questionnaire was used to measure cabin crews’ confidence related to the organizing and executing process of care during resuscitation. It was adapted from The Resuscitation Self-Efficacy Scale developed by Roh et al. [32] with four dimensions, namely Recognition, Debriefing and recording, Responding and rescuing, and Reporting. It contained 17 items and used a 5-point Likert scale ranging from 1 “least confident” to 5 “very confident.” The internal consistency coefficients of the four subscales were 0.82, 0.88, 0.87 and 0.83 respectively, and the overall Cronbach’s alpha was 0.91 by Roh et al. [32]. Altogether, the questionnaire of Self-Efficacy of First Aid (SEFA) contained 35 questions, a detailed description of the 7-subscale first aid capabilities that cabin crew feel confident of is presented below:

(1) Recognition & Alertness (of cardiac arrest): Performing an appropriate preliminary assessment during acute emergencies. (e.g., Shouting for help and continuing Primary Survey.)
(2) Cardiopulmonary Resuscitation: Performing chest compressions and rescue breaths in an emergency. (e.g., Effective chest compressions.)
(3) Safe use of an AED: Using a defibrillator (AED) to save lives. (e.g., Ensuring that nobody touches the victim.)
(4) Recognition (of emergency situations): Demonstrating correct measurement, interpreting and documenting vital signs. (e.g., Recognizing signs and symptoms of a critical event.)
(5) Debriefing & recording: Staying calm and focused on required tasks, completing quality improvement documentation. (e.g., Performing debriefing or problem solving after the event.)
(6) Responding & rescuing: Performing cardiopulmonary resuscitation according to resuscitation algorithm. (e.g., Demonstrating effective bag valve mask ventilations, i.e., volume, minute volume, pressure, etc.)
(7) Reporting: Providing appropriate messages and information to resuscitation team member. (e.g., Utilizing resources and external experts.)

#### 2.2.3. The Questionnaire of Work-Related Hardiness (WRH)

The third questionnaire was developed to assess cabin crews’ work-related hardiness. It was adapted from the Revised Academic Hardiness Scale by Benishek et al. [40], included four dimensions, commitment, control of effort, control of affect, and challenge. This scale included 39 items measured on a 5-point Likert scale ranging from 1 “strongly disagree” to 5 “strongly agree”. Cabin crew who scored higher on WRH indicate desirable levels of hardiness. The reliability of the four factors were 0.91, 0.91, 0.88, and 0.81, for the overall reliability, the Cronbach’s alpha value was 0.90 by Benishek et al. [40]. Each dimension includes 7 to 12 items. The definition of each factor is listed below [40].

The third questionnaire was developed to assess cabin crews’ work-related hardiness. It was adapted from the Revised Academic Hardiness Scale by Benishek et al. [41], including four dimensions, namely Commitment, Control of effort, Control of affect and Challenge. This instrument included 39 items measuring on a 5-point Likert scale ranging from 1 “strongly disagree” to 5 “strongly agree”. Cabin crew scoring higher on WRH indicates desirable levels of hardiness. The Cronbach’s alpha of the four factors were 0.91, 0.91, 0.88, and 0.81, and the overall reliability is 0.90 by Benishek et al. [40]. Each dimension includes 7 to 12 items. The definition of each factor is listed below [40].

(1) Commitment: Indicative of cabin crews’ willingness to put forth sustained effort and make sacrifices to excel work achievement (e.g., Won’t go out with friends if I need to prepare for recurrent training course).
(2) Control of effort: Associated with cabin crews’ ability to recognize and activate behaviors that increase their ability to overcome difficulties at work (e.g., Know when to ask for help).
(3) Control of affect: Representing cabin crews’ ability to regulate their emotions when faced with job challenges (e.g., Good at decreasing stress if not performing well at work).
(4) Challenge: Intent on seeking out difficult and stressful work and viewing these challenges as experiences that will ultimately contribute to their personal growth (e.g., Take difficult tasks at work because I know they will benefit me in long run).

### 2.3. Data Analyses

In the first stage, an exploratory factor analysis (EFA) was conducted in SPSS (version22.0, IBM Corp., Armonk, NY, USA) to identify the number of factors and the inner structure of the constructs, with a randomly selected sample of 207 participants using the randomization function on SPSS. Next, we used the remaining sample of 318 participants to examine the relationships among the dimensions of these three questionnaires (FAA, SEFA, and WRH). Using structural equation modeling (SEM) technique in AMOS, confirmatory factor analysis (CFA) was conducted to test the measurement model by including all the three questionnaires within one model; then correlation analysis in SPSS and path analysis using SEM were performed to examine the relationships among all constructs. To ensure the convergent validity of this proposed measurement model, three rules were followed: (1) all of the items’ standardized factor loadings in the CFA should be higher than 0.50; (2) the values of the composite reliability should exceed 0.70; and (3) the average variance extracted should exceed 0.50 [49,50]. Descriptive statistics were used to analyze the frequencies and means of the variables. Finally, the mediating roles of cabin crews’ WRH in the relationship between their FAA and SEFA were examined in this study. The bootstrap procedure in the AMOS software was used to analyze the direct relationship (with or without a mediator), the indirect relationship, and the mediation type. As *p* values are sensitive to the large sample size (*n* = 525), the level of statistical significance was raised to *p* < 0.001 to reduce the chances of a type I error [51,52].

### 2.4. Ethical Statement

Because of the observational nature of the study, and also in the absence of any involvement of therapeutic medication, no formal approval of the Institutional Review Board of the local Ethics Committee was required. Nonetheless, informed consent was obtained from all subjects involved in the study and all subjects were informed about the study and participation was fully on voluntary basis. The study was conducted in accordance with the Helsinki Declaration.

## 3. Results

In order to examine the underlying structure of FAA, WRH and SEFA in this study, EFA, CFA and reliability analysis were carried out using the data from each of the three questionnaires.

### 3.1. EFA for the Questionnaire of First Aid Affect (FAA)

To validate the questionnaire, an exploratory factor analysis (EFA) with principle component extraction and varimax rotation was performed to clarify its structure. Two factors were extracted, which accounted for 78.29% of the total variance. The means, standard deviations, reliability estimates, and the factor loadings for the retained items were presented in Table 1. The results of this EFA showed that all the FAA items loaded on the appropriate factor with factor loadings ranging from 0.65 to 0.94. The two factors were “Attitude (ATT)” (Mean = 4.59), “Willingness (WIL) (Mean = 3.07)”. The internal consistency coefficients of the two subscales were 0.89, and 0.95 respectively, and the overall Cronbach’s alpha was 0.85. Therefore, the FAA was deemed to be sufficiently reliable for assessing cabin crews’ first aid affect. 

### 3.2. EFA for the Questionnaire of Work-Related Hardiness (WRH)

The original scale of the Revised Academic Hardiness Scale (RAHS) generates four dimensions measuring hardiness. However, the results of the factor analysis in the current study revealed only three factors, explaining 71.86% of the total variance in the items. Table 2 contained the final 12-item, 3-factor scale. 

The first factor (labeled Commitment) included three items, and the Cronbach’s alpha was 0.77. The second factor (labeled Control) included six items, and the Cronbach’s alpha was 0.89. The third factor (labeled Challenge) included three items, and the Cronbach’s alpha was 0.86. and the overall Cronbach’s alpha for the final 12-item scale was 0.91. Moreover, the means ranged from 3.42 to 4.09. In sum, the WRH used in this study has high validity and sufficient reliability for assessing cabin crews’ work-related hardiness.

### 3.3. EFA for the Questionnaire of Self-Efficacy of First Aid (SEFA)

Based on the results of the EFA analysis, 16 items were retained, with 80.24% of variance explained. As shown in Table 3, the cabin crews’ responses to the SEFA can be grouped into the five factors: Recognition (three items), Debriefing & recording (three items), Responding & rescuing (three items), Reporting (three items), and Safe use of an AED (four items). The result is different from the original seven factors designed in SEFA. The internal consistency coefficients of the five subscales were 0.82, 0.82, 0.92, 0.88, and 0.92 respectively, and the overall Cronbach’s alpha was 0.94. The means ranged from 3.30 to 3.90. Therefore, the SEFA used in this study was sufficiently reliable for cabin crews’ first aid self-efficacy.

### 3.4. CFA Analyses for the Three Questionnaires

In order to clarify the reliability and validity of the research instrument, the present study conducted a single CFA with all the items and dimensions of the three questionnaires (FAA, WRH, and SEFA) recruited in one model. Based on the aforementioned criteria, a total of 38 items were retained in the final version (i.e., 10 items for FAA, 12 items for WRH, and 16 items for SEFA). In Table 4, three to four items were retained for each dimension. The reliability (Cronbach’s alpha) coefficients for all the dimensions ranged from 0.76 to 0.94, and the overall alpha is 0.82. The means ranged from 2.99 to 4.58, the composite reliability (CR) coefficients ranged from 0.78 to 0.95, all exceeding 0.70 [50]. The Average Variance Extracted (AVE) value of all factors ranged from 0.52 to 0.78, exceeding the cut-off value of 0.50. In addition, all the factor loadings of the measured items were statistically significant and higher than 0.5 [49].

The goodness of fit of the structure were obtained (χ^2^/df = 1.70 (χ^2^ = 1052.86, df = 620), *p* < 0.001, GFI = 0.86, TLI = 0.94, CFI = 0.95, IFI = 0.95, and RMSEA = 0.05), and all fell within the acceptable range [53,54,55,56], thus confirming the convergent and construct validity of this model for these three questionnaires.

### 3.5. Correlational Relationships among Different Dimensions

Correlation analysis was performed through the Pearson correlation analysis to determine whether the variables are mutually independent or related. The correlations among the dimensions of these three questionnaires, FAA, WRH, and SEFA, were presented in Table 5. The results indicated that Attitude in FAA was positively correlated with three SEFA dimensions: Responding and rescuing, Report and Safe use of an AED (r = 0.22~0.30, *p <* 0.001), whereas Willingness in FAA was positively correlated with all dimensions of SEFA (r = 0.22~0.30, *p <* 0.001).

In general, each dimension of the WRH was statistically significantly associated with each dimension of the SEFA (r = 0.22~0.52, *p* < 0.001), except for the correlation between WRH dimension CHA with SEFA dimension Safe use of an AED (r = 0.17, *p =* 0.003 > 0.001).

Positive correlations can be identified between FAA and two of WRH dimensions COM, CON (r = 0.23~0.29, *p <* 0.001). On the contrary, the correlation between FAA and WRH dimension CHA turned out to be not statistically significant (r = 0.08, *p* > 0.001; r = 0.11, *p* > 0.001).

### 3.6. The Structural Relationships among Cabin Crews’ First Aid Affect, Work-Related Hardiness and Self-Efficacy of First Aid

**Figure 2:**
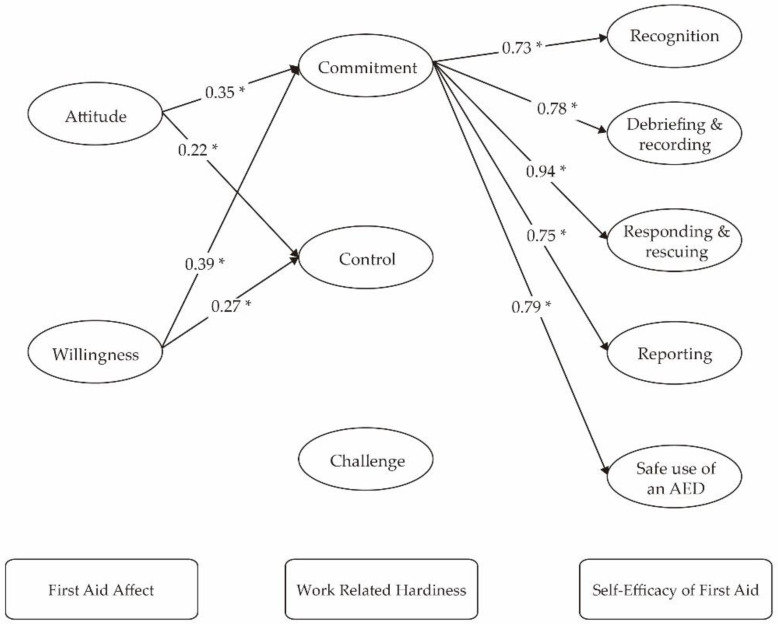
The structural equation model of the relationships among FAA, WRH, and SEFA. * *p* < 0.001.

We performed SEM analysis based on the correlation results. Figure 2. shows the structural relationships among the three questionnaires (FAA, WRH and SEFA). The fit indices revealed that the model adequately explained the data, (χ^2^/df = 2.33 (χ^2^ = 1475.11, df = 633), *p* < 0.001, GFI = 0.80, TLI = 0.89, CFI = 0.91, IFI= 0.91, and RMSEA = 0.07). The path with no statistical significance is omitted. Both Attitude and Willingness have positive associations with WRH Commitment, respectively (path coefficient *=* 0.35, *p* < 0.001; path coefficient = 0.39, *p* < 0.001). In addition, cabin crews’ WRH (i.e., Commitment) was highly correlated with all the 5 dimensions of SEFA respectively (Recognition: path coefficient = 0.73, *p* < 0.001; Debriefing and recording: path coefficient = 0.78, *p* < 0.001; Responding and rescuing: path coefficient = 0.94, *p* < 0.001; Reporting: path coefficient = 0.75, *p <* 0.001; Safe use of an AED: path coefficient = 0.79, *p <* 0.001). In other words, the associations between FAA and SEFA may be mediated through Commitment in WRH. Meanwhile, both Attitude and Willingness have positive associations with WRH (i.e., Control) respectively (path coefficient = 0.22, *p <* 0.001; path coefficient = 0.27, *p <* 0.001). The detailed results of the structural equation modeling assessment were shown in Table 6. The results imply that cabin crews with higher levels of attitudes and willingness toward first aid tend to have a stronger sense of professional and organizational identities, and they may have more confidence in their abilities to perform the first aid rescue. Thus, Hypotheses 1, 2 and 3 were partially supported.

### 3.7. Testing of Mediation

The SEM results indicated that FAA has a direct association with SEFA and an indirect association through WRH (i.e., Commitment). Thus, we evaluated whether the association between FAA and SEFA is mediated by WRH (i.e., Commitment). Some researchers claimed that significant direct effect between predictor and outcome before examining a mediator are not necessary [48,57,58]. Significance of the indirect effect should be only one requirement to establish mediation [48], so we followed the procedures of Zhao et al. [48] to evaluate whether WRH (i.e., Commitment) mediated the effect of FAA on SEFA. First, we tested the indirect path to determine whether WRH (i.e., Commitment) significantly mediated the association between FAA and SEFA. Second, we analyzed whether FAA and SEFA were significantly associated. After this, we estimated the mediated effect and direct effect to decide the mediation type of WRH (i.e., Commitment).

#### 3.7.1. Complementary Mediation

Results presented in Table 7 indicated that WRH (i.e., Commitment) has a complementary mediating effect on the relationship between Willingness in FAA and all the dimensions of SEFA. In other words, both the direct path and the indirect path existed in the same direction. The indirect paths from Willingness in FAA through Commitment to SEFA were all significant (*β* = 0.27~0.35, *p* < 0.001), and the direct path from Willingness in FAA to SEFA were also statistically significant (*β* = 0.26~0.35, *p* < 0.001). As the coefficients were all positive, the value of mediated effect and direct effect is positive and the complementary mediation is established. This means that if cabin crews are more willing to perform first aid, they are more committed to first aid, thus they hold a higher first aid self-efficacy. Meanwhile, Commitment in WRH also played a complementary mediating role between Attitude in FAA and Report and Safe use of an AED in SEFA. The results indicated that cabin crews’ work commitment partially mediated the relationships between their attitude toward first aid and their self-efficacy of first aid in REP and AED.

#### 3.7.2. Indirect-Only Mediation

As reported in Table 7, Commitment in WRH played an indirect-only mediating role in the relationship between Attitude in FAA and the first three factors of SEFA (i.e., Recognition, Debriefing & recording, Responding & rescuing). The indirect paths from FAA (i.e., Attitude) through WRH (i.e., Commitment) to SEFA (i.e., Recognition, Debriefing & recording, Responding & rescuing) were significant (*β* = 0.24~0.31, *p* < 0.001), whereas the direct path from FAA (i.e., Attitude) to SEFA (i.e., Recognition, Debriefing & recording, Responding & rescuing) were not significant (*β* = 0.11~0.89, *p* > 0.001). The results indicated that cabin crews’ attitude toward first aid predicted their self-efficacy of first aid (REC, DEB, RES) only when mediated by their commitment of work.

## 4. Discussion and Implication

The aim of the current study was to investigate the relationships among cabin crews’ first aid affect, work-related hardiness and, self-efficacy of first aid, including the testing of the mediating role of work-related hardiness.

### 4.1. Relationships between First Aid Affect and Self-Efficacy of First Aid

As expected, we found that crews’ first aid affect and most of the dimensions in self-efficacy of first aid were positively correlated, which means that cabin crews with better attitude and more willingness toward first aid may have more confidence in their abilities to handle the situation during medical emergencies. This is consistent with the results obtained in previous research [17,18,23] irrespective of different environments and with different group specifications. It also echoes Park et al.’s [59] statement that participants’ attitude and self-efficacy of CPR would affect their willingness to perform CPR. Therefore, it can be concluded that crews’ affective states play an essential role in their self-efficacy of first aid.

### 4.2. Relationships between Work-Related Hardiness and Self-Efficacy of First Aid

Close associations between hardiness and self-efficacy have been highlighted by previous researchers [43,44,45]. In this study, such relationships were also identified in the cabin crews’ work-related hardiness and their self-efficacy of first aid. Specifically, the path analysis of this study revealed that the crews’ work commitment was a positive predictor of their self-efficacy of first aid. This finding resonated with the previous studies on work commitment. For example, McKimet al. [60] found that teachers’ sense of efficacy in classroom management and science teaching was significantly related to teachers’ career commitment. Park et al. [61] reported that employees’ occupational self-efficacy was positively associated with career commitment and organizational commitment.

Aside from providing services to passengers in the cabin, the most important role of the cabin crew is to shoulder the responsibility of maintaining passengers’ safety should any emergency arise. Therefore, crew who are more willing to take the initiative to go all out for the team and make a full commitment to their job may have more confidence in handling in-flight medical emergencies.

Jang et al. [62] revealed that three dimensions of academic hardiness (i.e., commitment, control of affect, and challenge) were strong predictors of university students’ academic self-efficacy. Other studies also identified the relationship [43,63]. However, an interesting finding of our study was that the control and challenge dimensions of work-related hardiness were unrelated to first aid self-efficacy. One research from Sheard [64] found similar results in that only students’ commitment in their perseverance (hardiness) could positively predict their academic achievement. The results in our study may suggest that cabin crews who hold higher control over their effort and affect and are more willing to take the challenge tend to care more about their career goals. They are more focused on attaining recognition or promotions throughout their career, thus work harder to improve their professional skills. However, in some high-stakes situations, such as an in-flight medical emergency, they might be more concerned that a wrong treatment could have a serious impact on their career, leading to wavered self-efficacy of first aid.

### 4.3. Testing of Mediation

The findings in this study provided support for the mediating role played by work’s commitment in the relationship between cabin crew’s affect toward first aid and their self-efficacy of first aid. Specifically, the crews’ work commitment is an indirect-only mediator on the relationship between their willingness toward first aid and their self-efficacy of first aid. Consequently, enhancing cabin crew’s willingness toward first aid could be beneficial not only to building their work commitment, it could also lead to improved self-efficacy in performing first aid.

## 5. Practical Implications

Our research findings have important practical implications. To begin with, fostering crews’ positive affect towards first aid should be placed as one of the important goals of routine cabin crew training. This includes building cabin crew’s positive attitude toward first aid and developing their willingness to perform first aid on a variety of medical conditions. If cabin crews believe that performing first aid could have a positive impact on others and themselves, they would develop higher commitment toward their work, exert more control over their effort, and gain higher confidence in their capabilities of first aid.

To foster crews’ positive affect towards first aid is one of the important goals during routine crew training. Some previous studies have indicated that individuals with previous training experience and had a better understanding of CPR procedure were more willing to perform first aid. Besides, individuals with accurate knowledge about resuscitation and laws or legal policy statements have a more positive attitude toward first aid [22,25,28,29,65].

Our finding suggests that work-related commitment is a vital quality to be developed since it plays an important role in the relationship between cabin crews’ first aid affect and their self-efficacy of first aid. Ferreia [66] found significant relationships between the hardy-commitment and organizational commitment, suggesting that people who were deeply committed to whatever they deemed important and stay involved seem to emotionally attached to the organization. Airline companies should build positive organizational culture that supports cabin crew’s involvement, improves their work-related morale and stimulates their motivation. The airlines could conduct employee satisfaction surveys on an annual basis and ameliorate working environment with the ongoing feedback. Effective communication is helpful in increasing employee commitment. Plus, the airlines can create more career development opportunities, help employees build a clearer career path.

## 6. Limitation

First, this study is limited to correlational analysis among crews’ affect of first aid, work-related hardiness, and first aid self-efficacy, it cannot infer to causal relationships. Second, all data were based solely on self-reported questionnaires. It should be complemented by other methods such as interviews in the future.

## 7. Future Research

The current study found no association between self-efficacy of first aid and the control and challenge dimensions of work-related hardiness in crews. Since the cabin crews’ affect towards first aid is an independent variable in this study, future studies should build on these findings to explore additional factors. Further study is needed to continue searching for other potential mediators that may contribute to the relationship between first aid affect and self-efficacy of first aid.

Bandura [67] believes that self-efficacy influences how individuals feel, think, motivate themselves and behave. Thus, we used the cabin crew’s first aid self-efficacy as the main subject to discuss relationship with the affect towards first aid and work-related hardiness. Turner et al. [68] confirmed that there has a relationship between pediatricians’ confidence in their ability to perform first aid on children and respond to first aid. Future studies should focus on the association between the cabin crew’s self-efficacy and their performance towards first aid. Whether or not they can integrate their knowledge into work performance is also worth investigation.

## 8. Conclusions

To our knowledge, this study is the first to establish associations among cabin crew’s first aid affect, hardiness, and self-efficacy of first aid. 

This study determines that first aid affect and work-related hardiness are important factors relating to the cabin crews’ self-efficacy of first aid. In the present study, we have demonstrated that cabin crews with a positive attitude and more willingness toward first aid may have more confidence in their abilities to deal with medical emergencies. Likewise, the study also highlights cabin crews’ work commitment plays a mediating role between their first aid affect and self-efficacy of first aid.

These findings serve as an important reference for developing policy and practice to promote self-efficacy of first aid among cabin crews. 

## Figures and Tables

**Table 1:** Results of EFA for FAA (*N* = 207).

	Factor1 ATT	Factor2 WIL
Factor1: Attitude (ATT), Cronbach’s alpha = 0.89, Mean = 4.59, SD = 0.51		
ATT 1	0.87	
ATT 2	0.89	
ATT 3	0.90	
ATT 4	0.93	
ATT 5	0.65	
Factor2: Willingness (WIL), Cronbach’s alpha = 0.95, Mean = 3.07, SD = 1.14		
WIL 1		0.79
WIL 2		0.92
WIL 3		0.94
WIL 4		0.94
WIL 5		0.94

Actual form total variance explained: 78.29%, overall alpha: 0.85.

**Table 2:** Results of EFA for WRH (*N* = 207).

	Factor1 COM	Factor2 CON	Factor3 CHA
Factor1: Commitment (COM), Cronbach’s alpha = 0.77, Mean = 4.09, SD = 0.58			
COM 1	0.69		
COM 2	0.78		
COM 3	0.82		
Factor2: Control (CON), Cronbach’s alpha = 0.89, Mean = 3.99, SD = 0.56			
CON1		0.58	
CON2		0.76	
CON3		0.75	
CON4		0.74	
CON5		0.73	
CON6		0.66	
Factor3: Challenge (CHA), Cronbach’s alpha = 0.86, Mean = 3.42, SD = 0.86			
CHA1			0.82
CHA2			0.84
CHA3			0.80

Actual form total variance explained: 71.86%, overall alpha: 0.91.

**Table 3:** Results of EFA for SEFA (*N* = 207).

	Factor1 REC	Factor2 DEB	Factor3 RES	Factor4 REP	Factor5 AED
Factor1: Recognition (REC), Cronbach’s alpha = 0.82, Mean = 3.30, SD = 0.72					
REC1	0.78				
REC2	0.74				
REC3	0.81				
Factor2: Debriefing & recording (DEB), Cronbach’s alpha = 0.82, Mean = 3.45, SD = 0.70					
DEB1		0.68			
DEB2		0.74			
DEB3		0.63			
Factor3: Responding & rescuing (RES), Cronbach’s alpha = 0.92, Mean = 3.52, SD = 0.77					
RES1			0.80		
RES2			0.79		
RES3			0.68		
Factor4: Reporting (REP), Cronbach’s alpha = 0.88, Mean = 3.72, SD = 0.69					
REP1				0.85	
REP2				0.86	
REP3				0.61	
Factor5: Safe use of an AED (AED), Cronbach’s alpha = 0.92, Mean = 3.90, SD = 0.67					
AED1					0.83
AED2					0.87
AED3					0.85
AED4					0.68

Actual form total variance explained: 80.24%, overall alpha: 0.94.

**Table 4:** The CFA analysis of the three separate questionnaires (FAA, WRH and SEFA) (*N* = 318).

Construct and Measurement Items		Factor Loading	t	AVE	CR	Alpha Value	Mean	SD
The questionnaire of first aid affect (FAA)								
Attitude (ATT)		-	-	0.77	0.95	0.94	4.58	0.61
ATT 1	Having knowledge and skills in first aid is very helpful and important to my family.	0.85	-					
ATT 2	If I can give first aid to others in time, saving a life can be very meaningful.	0.93	22.77 *					
ATT 3	Proper first aid can not only save lives and prevent the injury from getting severe; it can also help the injured recover.	0.90	21.68 *					
ATT 4	Having first aid knowledge and skills can save lives.	0.93	23.10 *					
ATT 5	Everyone should have basic first aid knowledge.	0.78	16.93 *					
Willingness (WIL)		-	-	0.76	0.94	0.94	2.99	1.13
WIL 1	I’m willing to perform first aid for emergency childbirth.	0.68	-					
WIL 2	I’m willing to perform first aid on a hyperventilating victim.	0.90	14.87 *					
WIL 3	I’m willing to perform first aid on a choking victim.	0.92	15.06 *					
WIL 4	I’m willing to perform first aid on a seizure victim.	0.94	15.36 *					
WIL 5	I’m willing to perform first aid on a syncope victim.	0.90	14.72 *					
The questionnaire of work-related hardiness (WRH)								
Commitment (COM)		-	-	0.54	0.78	0.77	4.08	0.59
COM 1	I put effort into all works.	0.71	-					
COM 2	I do my best irrespective of the task given.	0.72	11.35 *					
COM 3	Doing well in job is as important to me as to managers and colleagues.	0.77	12.03 *					
Control (CON)		-	-	0.56	0.88	0.88	3.98	0.57
CON 1	With hard work, I can reach my career goals.	0.66	-					
CON 2	I can stay calm and learn from mistakes at work.	0.81	12.40 *					
CON 3	I do not give up when not doing well or meeting difficult situations at work.	0.80	12.25 *					
CON 4	I’m good at calming myself and seeking help from others when performing poorly at work.	0.82	12.50 *					
CON 5	I’m good at reducing stress if not performing well at work.	0.72	11.20 *					
CON 6	I can manage stress from difficult job in healthy ways.	0.65	10.26 *					
Challenge (CHA)		-	-	0.63	0.83	0.83	3.31	0.81
CHA 1	As long as I am interested, I’m willing to challenge the difficult job, and risk being blamed or complained.	0.76	-					
CHA 2	I take challenging tasks at work that promotes career growth.	0.87	13.90 *					
CHA 3	I take the initiative to propose new ideas to the management at work.	0.73	12.47 *					
The questionnaire of self-Efficacy of first aid (SEFA)								
Recognition (REC)		-	-	0.52	0.77	0.76	3.28	0.67
REC 1	I’m confident that I can demonstrate correct measurement, interpretation and documentation of vital signs.	0.73	-					
REC 2	I’m confident that I can recognize signs and symptoms of a critical event.	0.72	10.59 *					
REC 3	I’m confident that I can demonstrate a focused assessment following the ABC (Airway, Breathing, Circulation) principles.	0.72	10.58 *					
Debriefing and recording (DEB)		-	-	0.64	0.84	0.84	3.35	0.71
DEB 1	I’m confident that I can perform debriefing or problem solving after the event.	0.80	-					
DEB 2	I’m confident that I can complete quality improvement documentation.	0.83	15.13 *					
DEB 3	I’m confident that I can demonstrate staying calm and focusing on required tasks.	0.76	13.84 *					
Responding and rescuing (RES)		-	-	0.78	0.91	0.90	3.52	0.75
RES 1	I’m confident that I can perform CPR according current guidelines.	0.96	-					
RES 2	I’m confident that I can conduct effective chest compressions.	0.94	31.37 *					
RES 3	I’m confident that I can conduct effective rescue breaths.	0.73	17.44 *					
Reporting (REP)		-	-	0.65	0.84	0.84	3.69	0.63
REP 1	I’m confident that I can utilize resources and external experts.	0.85	-					
REP 2	I’m confident that I can demonstrate the use of appropriate means of communication guided by the company’s policy.	0.85	16.87 *					
REP 3	I’m confident that I can understand when to call for help.	0.70	13.44 *					
Safe use of AED (AED)		-	-	0.78	0.93	0.93	3.87	0.65
AED 1	I’m confident that I can switch AED on and use it as soon as it becomes available.	0.86	-					
AED 2	I’m confident that I can follow AED prompts in the right order without confusion or distraction.	0.90	21.64 *					
AED 3	I’m confident that I can attach AED pads to the correct positions.	0.92	22.91 *					
AED 4	I’m confident that I can proceed as directed from AED prompts.	0.85	19.75 *					

Notes: * *p* < 0.001, overall alpha: 0.82, AVE: average variance extracted, CR: composite reliability.

**Table 5:** The correlations among the dimensions of the FAA, WRH, and SEFA.

	ATT	WIL	COM	CON	CHA	REC	DEB	RES	REP	AED
ATT	1									
WIL	0.17	1								
COM	0.28 *	0.29 *	1							
CON	0.23 *	0.27 *	0.68 *	1						
CHA	0.08	0.11	0.43 *	0.53 *	1					
REC	0.14	0.22 *	0.31 *	0.34 *	0.22 *	1				
DEB	0.15	0.30 *	0.46 *	0.52 *	0.34 *	0.50 *	1			
RES	0.19 *	0.30 *	0.44 *	0.38 *	0.24 *	0.55 *	0.57 *	1		
REP	0.23 *	0.27 *	0.48 *	0.49 *	0.28 *	0.38 *	0.59 *	0.56 *	1	
AED	0.23 *	0.26 *	0.38 *	0.35 *	0.17	0.35 *	0.42 *	0.63 *	0.55 *	1

Notes: * *p* < 0.001, FAA dimensions: ATT: Attitude; WIL: Willingness; WRH dimensions: COM: Commitment; CON: Control; CHA: Challenge; SEFA dimensions: REC: Recognition; DEB: Debriefing and recording; RES: Responding and rescuing; REP: Reporting; AED: Safe use of an AED.

**Table 6:** Result of the structural model evaluation.

Hypotheses	Path	Coefficients	Status
Hypothesis 1	First aid affect → Self-efficacy of first aid		
Hypothesis 1.1	First aid affect (Attitude) → Self-efficacy of first aid (Recognition)	−0.15	Not Supported
Hypothesis 1.2	First aid affect (Attitude) → Self-efficacy of first aid (Debriefing and recording)	−0.23	Not Supported
Hypothesis 1.3	First aid affect (Attitude) → Self-efficacy of first aid (Responding and rescuing)	−0.17	Not Supported
Hypothesis 1.4	First aid affect (Attitude) → Self-efficacy of first aid (Reporting)	−0.09	Not Supported
Hypothesis 1.5	First aid affect (Attitude) → Self-efficacy of first aid (Safe use of AED)	−0.07	Not Supported
Hypothesis 1.6	First aid affect (Willingness) → Self-efficacy of first aid (Recognition)	−0.03	Not Supported
Hypothesis 1.7	First aid affect (Willingness) → Self-efficacy of first aid (Debriefing and recording)	−0.01	Not Supported
Hypothesis 1.8	First aid affect (Willingness) → Self-efficacy of first aid (Responding and rescuing)	−0.06	Not Supported
Hypothesis 1.9	First aid affect (Willingness) → Self-efficacy of first aid (Reporting)	−0.06	Not Supported
Hypothesis 1.10	First aid affect (Willingness) → Self-efficacy of first aid (Safe use of AED)	−0.05	Not Supported
Hypothesis 2	First aid affect → Work hardiness		
Hypothesis 2.1	First aid affect (Attitude) → Work hardiness (Commitment)	0.35 *	Supported
Hypothesis 2.2	First aid affect (Attitude) → Work hardiness (Control)	0.22 *	Supported
Hypothesis 2.3	First aid affect (Attitude) → Work hardiness (Challenge)	0.07	Not Supported
Hypothesis 2.4	First aid affect (Willingness) → Work hardiness (Commitment)	0.39 *	Supported
Hypothesis 2.5	First aid affect (Willingness) → Work hardiness (Control)	0.27 *	Supported
Hypothesis 2.6	First aid affect (Willingness) → Work hardiness (Challenge)	0.13	Not Supported
Hypothesis 3	Work hardiness → Self-efficacy of first aid		
Hypothesis 3.1	Work hardiness (Commitment) → Self-efficacy of first aid (Recognition)	0.73 *	Supported
Hypothesis 3.2	Work hardiness (Commitment) → Self-efficacy of first aid (Debriefing and recording)	0.78 *	Supported
Hypothesis 3.3	Work hardiness (Commitment) → Self-efficacy of first aid (Responding and rescuing)	0.94 *	Supported
Hypothesis 3.4	Work hardiness (Commitment) → Self-efficacy of first aid (Reporting)	0.75 *	Supported
Hypothesis 3.5	Work hardiness (Commitment) → Self-efficacy of first aid (Safe use of AED)	0.79 *	Supported
Hypothesis 3.6	Work hardiness (Control) → Self-efficacy of first aid (Recognition)	−0.01	Not Supported
Hypothesis 3.7	Work hardiness (Control) → Self-efficacy of first aid (Debriefing and recording)	0.19	Not Supported
Hypothesis 3.8	Work hardiness (Control) → Self-efficacy of first aid (Responding and rescuing)	−0.10	Not Supported
Hypothesis 3.9	Work hardiness (Control) → Self-efficacy of first aid (Reporting)	0.16	Not Supported
Hypothesis 3.10	Work hardiness (Control) → Self-efficacy of first aid (Safe use of AED)	−0.05	Not Supported
Hypothesis 3.11	Work hardiness (Challenge) → Self-efficacy of first aid (Recognition)	0.00	Not Supported
Hypothesis 3.12	Work hardiness (Challenge) → Self-efficacy of first aid (Debriefing and recording)	0.03	Not Supported
Hypothesis 3.13	Work hardiness (Challenge) → Self-efficacy of first aid (Responding and rescuing)	−0.06	Not Supported
Hypothesis 3.14	Work hardiness (Challenge) → Self-efficacy of first aid (Reporting)	−0.04	Not Supported
Hypothesis 3.15	Work hardiness (Challenge) → Self-efficacy of first aid (Safe use of AED)	−0.09	Not Supported

Notes: * *p* < 0.001.

**Table 7:** Mediation tests.

Tested Relationship	Direct Model	Indirect Model	Result
Attitude → Commitment → Recognition	0.11	0.24 *	Indirect-only (Mediation)
Attitude → Commitment → Debriefing & recording	0.09	0.26 *	Indirect-only (Mediation)
Attitude → Commitment → Responding & rescuing	0.15	0.31 *	Indirect-only (Mediation)
Attitude → Commitment → Reporting	0.20 *	0.25 *	Complementary (Mediation)
Attitude → Commitment → Safe use of an AED	0.20 *	0.26 *	Complementary (Mediation)
Willingness → Commitment → Recognition	0.27 *	0.27 *	Complementary (Mediation)
Willingness → Commitment → Debriefing & recording	0.35 *	0.30 *	Complementary (Mediation)
Willingness → Commitment → Responding & rescuing	0.31 *	0.35 *	Complementary (Mediation)
Willingness → Commitment → Reporting	0.28 *	0.28 *	Complementary (Mediation)
Willingness → Commitment → Safe use of an AED	0.26 *	0.29 *	Complementary (Mediation)

Notes: * *p* < 0.001.

## Data Availability

Data are available from the corresponding author on reasonable request.
